# Prevalence of depression, anxiety, stress and its relationship with knowledge about COVID-19 in medical and laboratory medicine students of Umm-Al-Qura University: a cross-sectional survey

**DOI:** 10.1186/s41983-022-00590-7

**Published:** 2022-12-14

**Authors:** Sadia Sultan, MD. Abu Bashar, Aisha Tabassum, Mohammad Shahid Iqbal, Ibtesam Nomani, Nouf M. Almasoudi, Mawaddah Tayeb, Ghadi W. Munshi, Rahaf K. Matyuri

**Affiliations:** 1 Clinical Science Department, Fakeeh College for Medical Sciences, Jeddah, Saudi Arabia; 2 Department of Community Medicine and Family Medicine, AIIMS, Gorakhpur, U.P. India; 3 Department of Laboratory Medicine, Umm-Al-Qura University, Mecca, Saudi Arabia; 4 Department of Nursing Practices, Faculty of Nursing, Umm Al-Qura University, Mecca, Saudi Arabia

**Keywords:** COVID-19, Mental health, Anxiety, Depression, Stress

## Abstract

**Introduction:**

Coronavirus disease 2019 (COVID-19) pandemic has continued relentlessly for over one and half years now, causing a threat to life, fear of falling sick, helplessness, anxiety, depression and, pessimism about the future. There has been an increasing concern over student mental health in higher education. Our study was designed to measure current mental health status and its relationship with sociodemographic variables and level of knowledge about COVID-19 in Saudi Arabia.

**Method:**

A cross-sectional survey was conducted among laboratory medicine students of Makkah city, Saudi Arabia from October, 2020 to January, 21. A semi-structured questionnaire was circulated through mail and What’s App. Data collected included sociodemographic details and level of knowledge towards the COVID-19 among the students. Depression anxiety and stress-21 item (DASS 21) was used to assess psychological status.

**Result:**

Our study reported 51.4% depressive symptoms, 57.9% anxiety symptoms, and 48.5% stress in the study participants. History of being hospitalized with COVID-19 and ICU reported high anxiety (*p* = 0.0003) and depression scores (*p* = 0.04). Respiratory droplet as a mode of transmission revealed higher scores on anxiety subscale (*p* = 0.007), whereas surface contamination reported high score of stress (*p* = 0.004) and anxiety (*p* = 0.002). Knowing that COVID-19 can also clinically present with gastrointestinal symptoms was found to show high stress (*p* = 0.005) and anxiety (*p* = 0.01) scores than any other way of clinical presentation.

**Conclusion:**

COVID-19 is likely to cause negative effect on the psychological health of students.

## Introduction

The novel coronavirus disease 2019 (“COVID-19”) has gripped the world, causing alarm and dread across the world. Since the start of the pandemic through December 1st, 2020, there have been 347,157 confirmed (Covid-19) cases in Saudi Arabia, with 5907 fatalities [1]. Most cross-sectional surveys [2–6] of the general population showed increased symptoms of depression, anxiety, and stress-related to “COVID-19” due to its lethality, drastic change in lifestyle, social isolation, economic burden and, pessimism about the future, fear and worry about contracting the illness [7]. Studies from the Kingdom of Saudi Arabia reported high levels of stress, anxiety and, depression among the general population [8–10] and in particular university students [10, 11] during the pandemic. A survey of the mental health of college students during the 2019–2020 academic year, both before and after the COVID-19 pandemic, found a startlingly high prevalence of depression and anxiety [11].

Health education transition from face-to-face sessions to video lectures or live streams [12] lead to the stress and anxiety [13]. Although lecture-based teaching is simply transitioned to an online format, clinical exposure and providing authentic patient experiences are not as easily replicated. Such circumstances have an adverse effect on the mental health of medical and paramedical students. Pre-pandemic evidence suggests that medical and paramedical students have reported higher levels of perceived stress, anxiety, and depression than general population [13], which further increased during the pandemic. Increased anxiety, depression [14, 15] PTSD [16] and stress [17] symptoms were found in college and university students. Although the institutional response to “COVID-19” has been rapid, with a commitment to deliver academic services uninterruptedly, such a sudden change may cause more stress for students who are already under high levels of academic stress [18].

Adequate knowledge about pandemic may show reduced negative impact (anxiety and stress) in vulnerable population [19, 20]. Recognizing the psychological impact of the “COVID-19” outbreak in the general population and high-risk population like students and health care workers is pivotal in guiding policymakers and formulating interventions to maintain positive mental health. Reliable information about mental health changes associated with the pandemic will help service providers make decisions that are underpinned by knowledge of the scale of changes in population mental health and recognize who is most vulnerable to symptoms of mental distress. This study aimed to investigate the psychological outcome of “COVID-19” in university students. Furthermore, an association of these outcomes with sociodemographic variables and knowledge factors were determined.

## Methods

### Participants

A cross-sectional study through an online survey was conducted at Umm-Al-Qura University between October 1st to December 1st, 2020. The questionnaires were distributed to the target sample of university students through social media platforms using snowball-sampling technique. Platforms including Facebook, WhatsApp, and Twitter, as well as personal e-mail, were used for the recruitment and dissemination. The participants of this survey were mainly young people aged 18–25 years.

We calculated the sample size based on the assumption prevalence of 24% psychological impact according to Alkhamees et al. [10] at 5% absolute precision, power at 80% and a confidence interval of 95%. Taking into consideration a 20% non-response rate, the minimal sample size required was 345 participants. However, we intended to send the study questionnaires to 400 potential participants.

The inclusion criteria were current university students in Saudi Arabia who spoke and understood English and had access to the online questionnaire.

### Measures

#### Data collection sheet

A semi-structured study questionnaire was developed by researchers to obtain general information of the participants as such as gender, age, education, marital status, occupation, income. The Latter half of the questionnaire included questions related to general and mental health, “COVID 19” exposure and, knowledge regarding “COVID-19”. Knowledge questions included ten items inquiring about knowledge regarding mode of spread, ways of prevention and, symptoms. The participants could select “yes or no” options. A pilot study was done on 50 participants to check the validity and reality of the scale.

#### Depression Anxiety Stress Scale-21

Psychological outcomes were assessed using Depression Anxiety Stress Scales (DASS-21) The DASS-21 is a screening tool used for the general population for screening depression, anxiety and, stress. It is a self-administered 21-item instrument, which screens for depression, anxiety and, stress based on the recommended severity thresholds for the depression, anxiety, stress subscales [21]. Each subscale is composed of seven items, and each response was rated from 0 to 3. The depression subscales, scores of 0–9 was considered “normal” 10–12 “mild”, 13–20 “moderate”, 21–27 “severe”, and 28–42 as “extremely severe”. The anxiety sub-score was categorized into the following scores, “normal” (0–6) “mild” (7–9), “moderate” (10–14), “severe” (15–19), and extremely severe” (20–42). The stress subscale score was categorized into “normal” (0–10) “mild” (11–18), “moderate” (19–25), “severe” (26–33), and “extremely severe” stress [22]. Participants were asked to report the presence of a symptom over the past week. Scores for three emotional states were calculated by adding the points for the relevant items question for depression, stress, anxiety and doubling up the score [22].

### Statistical analysis

All the data collected through google forms were converted to excel spreadsheets and analysis was performed using IBM SPSS Statistics for Windows, version 22.0 (IBM Corp., Armonk, NY, USA). The data were cleaned, sorted, and processed before the commencement of analyses. The survey’s answers fields were designed to be mandatory to be filled before proceeding to the next section, options such as “None” or “I don’t know” were provided wherever necessary to minimize missed data. Kolmogorov–Smirnov (K–S) test was used to check normality of the data distribution.

Descriptive analyses were performed for sample characteristics, history of “COVID-19” in self or family, history of contact with “COVID-19” positive patient, knowledge towards various aspects of “COVID-19” and concerns, and precautionary measures. The results of these analyses were presented as frequencies and percentages for categorical variables and as means and standard deviations for continuous variables.

The psychological burden of the “COVID-19” pandemic was measured using scores on the three subscales of the DASS; the scores were presented as means with standard deviations. Bivariate analyses were performed to find the relationship between the individual variables and scores on each of the three scales (DASS-stress, DASS anxiety, and DASS-depression) using independent *t*-test to compare two groups. All tests of associations were carried out at a significance level of < 0.05.

## Results

### Participant’s characteristics

Out of the 400 students who were invited to participate in the survey through the online link, 369 agreed to participate and completed the study questionnaire giving a response rate of 92.2%. Among the participants 269 (72.8%) belonged to department of laboratory medicine and 100 (27.1%) to department of medicine. The demographic information for the 369 participants (323 [87.5%] females and 46 [12.5%] males) is shown in Table 1. Their ages ranged from 18 to 30 years but most of the participants were between 18 and 25 years (91.3%). Besides, 91.8% of the participant were undergraduate students and 8.2% were post-graduate students. Most of the participants were unmarried (91.3%). History of psychiatric illness was found in 14.6% and 18.7% reported a positive family history of psychiatric illness. The “COVID 19” exposure information revealed 16% got infected with the virus, 40.1% reported family members getting infected, 30.9% reported, quarantine due to contact with the positive patient and, 15.7% reported hospitalization with 24.4% landing up in the intensive care unit (ICU) (Table 1).

**Table 1:** Socio-demographic characteristics of the study participants (*N* = 369)

Variables	Frequency	Percentages
Sex		
Male	46	12.5
Female	323	87.5
Age-groups		
18–25	337	91.3
26–30	32	8.7
Level of education		
Undergraduate	346	91.8
Postgraduate	23	8.2
Marital status		
Unmarried	337	91.3
Married	24	6.5
Widowed/divorced/separated	08	2.2
History of psychiatric illness		
Present	69	14.6
Absent	315	85.4
Family history of psychiatric illness		
Present	69	18.7
Absent	300	81.3
History of any chronic physical illness		
Present	91	24.7
Absent	278	75.3
History of contracting COVID-19		
Yes	59	16.0
No	310	84.0
History of family member contracting COVID-19		
Yes	148	40.1
No	221	59.9
History of quarantine due to contact with positive patient		
Yes	114	30.9
No	255	69.1
History of close contact with COVID-19 patient in recent past		
Yes	124	33.6
No	245	66.4
History of hospitalization due to COVID 19 (yourself or any of family member)		
Yes	58	15.7
No	311	84.3
History of ICU admission (you or your family member)		
Yes	90	24.4
No	279	75.6

With respect to the knowledge on various aspects of COVID-19, majority of the participants knew correctly that COVID-19 can be prevented by use of face mask, social distancing, and hand washing (94.6%) and COVID-19 is mainly transmitted through respiratory droplets such as coughing and sneezing (94.0), whereas only 39.6% of the participants knew that 56° for 30 min, diethyl ether, 75% ethanol, peracetic acid and other lipid solvents can effectively kill the virus causing COVID

### Knowledge regarding “COVID-19” among students

Regarding the knowledge on various aspects of “COVID-19”, the majority (94.6%) of the participants knew correctly that “COVID-19” can be prevented by the use of face mask, social distancing, and handwashing and 94.0% knew its mode of transmission, whereas only 39.6% of the participants knew that 56 °C for 30 min, diethyl ether, 75% ethanol, peracetic acid and, other lipid solvents can effectively kill the virus causing “COVID-19”. The Majority of the participants exhibited good knowledge about general symptoms related to “COVID-19” illness (91.6%) including the knowledge about breathlessness and low oxygen saturation (85.9%), whereas 74.3% of the participants knew about gastrointestinal symptoms as the presentation of “COVID-19”. Awareness about psychological balance and its relation with improved immunity was found in 77.8% (Table 2).

**Table 2:** Responses to knowledge and practices questions on COVID-19 by the study participant (*N* = 369)

Sl. no.	Questions	No. of participant knowing correctly *N* (%)
1.	Do you know that 56° for 30 min, diethyl ether, 75% ethanol, peracetic acid and other lipid solvents can effectively kill the virus causing COVID?	146 (39.6)
2.	COVID-19’s incubation period is generally 3–7 days, with a maximum of 14 days	335 (90.8)
3.	COVID-19 is mainly transmitted through respiratory droplets such as coughing and sneezing	347 (94.0)
4.	COVID-19 is also transmitted through contact with contaminated surface and subsequently touching face	345 (93.5%)
5.	COVID-19 can be prevented by use of face mask, social distancing, and hand washing	349 (94.6)
6.	The early general symptoms of COVID-19 are fever, fatigue, dry cough, and gradually breathlessness. Some patients have mild onset symptoms without fever	338 (91.6)
7.	In severe cases, severe breathlessness with low oxygen in blood is seen	317 (85.9)
8.	Do you know it can also present with gastrointestinal symptoms such as diarrhea, vomiting and abdominal pain	274 (74.3)
9.	Do you know that psychological balance can improve the body’s immunity	287 (77.8)
10.	Do you keep check of number of cases and deaths due to COVID 19 in the city	186 (50.4)

As per DASS-21 scores of the participants, 190 (48.6%) were found to have depression, 214 (54.7%) were found to have anxiety and 179 (45.8%) were found to have stress. The classification of these participants into mild, moderate, severe, and very severe categories is shown in Table 3

### Psychological impact

Using the specified cut-offs of the DASS-21 scoring system to screen for depression, anxiety and, stress. We found depressive symptoms in 190 (51.4%), anxiety symptoms in 214 (57.9%) and, stress in 179 (48.5%) study participants. The overall mean DASS-21 depression subscale score was 11.25 ± 10.86. Among the 190 students who screened positive for depressive symptoms, 22.6% scored mild, 38.4% moderate, 15.8% severe and, 23.2% extremely severe on the DASS sub-score for depressive symptoms. The overall mean DASS-21 anxiety symptoms subscale score was 10.16 ± 9.62. Of the 214 patients who screened positive for anxiety, 13.6% of them showed mild anxiety, 38.8% moderate, 11.2% severe and, 36.4% extremely severe anxiety symptoms. In terms of the DASS-21 stress subscale, the overall mean score was 11.55 ± 10.36. Stress was mild in 49.7%, moderate in 27.9%, severe in 16.2% and, extremely severe in 6.2% participants screened positive for stress (*n* = 179) (Table 3).

**Table 3:** Prevalence and severity of depression, anxiety and stress among the study participants as per DASS-21 scores (*N* = 369)

Variables	DASS depression		DASS anxiety		DASS stress	
Score, mean ± SD	11.25 ± 10.86		10.16 ± 9.62		11.55 ± 10.36	
Categories, *N* (%)						
Mild	43	22.6	29	13.6	89	49.7
Moderate	73	38.4	83	38.8	50	27.9
Severe	30	15.8	24	11.2	29	16.2
Extremely severe	44	23.2	78	36.4	11	6.2
Total	190 (51.4%)	100.0	214 (57.9%)	100.0	179 (48.5%)	100.0

### Sample characteristics and psychological impact

There was no significant statistical association found between the DASS-21 score and sociodemographic factors like age, sex, marital status and, educational levels. The history of psychiatric illness in the participants showed higher scores on depressive symptoms (*p* = 0.001), anxiety symptoms (*p* = 0.01) and, stress (*p* = 0.04) symptoms. Positive family history of psychiatric illness was significantly related with a high score on depressive symptoms (*p* = 0.03), anxiety (*p* = 0.005) and, stress (*p* = 0.02) symptoms. While the history of chronic medical illness has shown high scores of stress symptoms (*p* = 0.01). History of contracting “COVID-19” was related with high scores on anxiety (*p* = 0.004) and depressive symptoms (*p* = 0.02) subscale while the factors such as family member getting infected with “COVID-19”, history of quarantine and close contact with “COVID”-positive case had not shown any significant change in DASS-21 scores. Participants hospitalized with “COVID-19” has shown a significantly high anxiety symptom (*p* = 0.0003). Participants admitted to ICU reported significantly high anxiety scores (*p* = 0.0003) and stress scores (*p* = 0.04) (Table 4).

**Table 4:** Association of DASS-Depression, Anxiety and Stress Scores with sociodemographic characteristics and COVID-19 contracting history of the participants (*N* = 369)

Variables	DASS Stress Score		DASS Anxiety Score		DASS Depression Score	
	Mean ± SD	*p* value	Mean ± SD	*p* value	Mean ± SD	*p* value
Gender						
Male	11.95 ± 10.39	0.89	10.69 ± 9.72	0.95	11.30 ± 9.61	0.91
Female	12.17 ± 10.28		10.79 ± 9.60		11.58 ± 9.72	
Age-group (in years)						
18–24	11.98 ± 10.30	0.30	10.55 ± 9.60	0.13	11.60 ± 9.72	0.34
25–30	13.94 ± 10.35		13.19 ± 9.64		10.94 ± 9.72	
Education status						
Undergraduate/graduate	12.14 ± 10.35	0.94	10.77 ± 9.67	0.91	11.56 ± 9.72	0.78
Postgraduate	12.26 ± 10.35		11.00 ± 9.64		11.30 ± 9.61	
Marital status				0.54		
Single/divorced/separated	12.14 ± 10.30	0.95	10.70 ± 9.61		11.49 ± 10.91	0.12
Currently married	12.25 ± 10.29		11.92 ± 9.58		12.25 ± 10.96	
History of psychiatric illness						
Absent	11.73 ± 10.30	0.04*	10.26 ± 9.60	0.01*	11.34 ± 9.72	0.001
Present	14.59 ± 10.67		13.81 ± 9.77		13.34 ± 10.97	*
Family history of psychiatric illness						
Absent	11.55 ± 10.30	0.02*	10.11 ± 9.60	0.005*	10.64 ± 9.72	0.03*
Present	14.75 ± 10.31		13.68 ± 9.62		11.74 ± 10.91	
History of any chronic illness						
Absent	11.40 ± 10.31	0.01*	10.24 ± 9.60	0.06	11.65 ± 10.94	0.89
Present	14.44 ± 10.32		12.41 ± 9.63		11.21 ± 10.92	
History of contracting COVID-19						
Absent	11.85 ± 10.30	0.20	10.14 ± 9.61	0.004*	10.54 ± 9.72	0.02*
Present	13.72 ± 10.40		14.10 ± 9.70		11.73 ± 10.89	
History of Family member contracting COVID-19						
Absent	11.77 ± 10.33	0.38	10.27 ± 9.60	0.21	11.40 ± 10.89	0.56
Present	12.72 ± 10.31		11.54 ± 9.61		11.76 ± 10.90	
History of quarantine due to contact with positive patient						
Absent	12.03 ± 10.30	0.73	10.42 ± 9.60	0.28	11.98 ± 10.90	0.34
Present	12.42 ± 10.41		11.58 ± 9.64		10.56 ± 11.00	
History of close contact with COVID-19 patient in recent past						
Absent	12.56 ± 10.31	0.53	10.10 ± 9.60	0.06	11.85 ± 10.90	0.78
Present	12.15 ± 10.33		12.11 ± 9.64		10.94 ± 10.93	
History of hospitalization						0.87
Absent	11.77 ± 10.30	0.10	9.99 ± 9.60	0.0003*	11.58 ± 10.90	
Present	14.17 ± 10.37		15.03 ± 9.68		11.34 ± 10.92	
History of ICU admission						0.18
Absent	11.56 ± 10.31	0.04*	9.74 ± 9.59	0.0003*	10.22 ± 10.89	
Present	13.98 ± 10.30		14.00 ± 9.60		11.97 ± 10.90	

*Statistically significant

### Knowledge variables and psychological impact

Most of the knowledge variables were not associated with significantly increased scores on DASS-21. The knowledge about the agents that can effectively kill the virus causing “COVID-19” showed significantly high scores on the depression subscale (*p* = 0.01) and respiratory droplet as a mode of transmission was associated with higher scores on the anxiety subscale (*p* = 0.007). Knowledge regarding the surface contamination as a mode of spread was associated with a significantly high score of stress (*p* = 0.004) and anxiety (*p* = 0.002). Knowing that “COVID-19” can also clinically present with gastrointestinal symptoms showed significantly high scores on stress (*p* = 0.005) and anxiety (*p* = 0.01) subscale than any other way of clinical presentation (Table 5).

**Table 5:** Association of knowledge of COVID-19 with DASS stress, anxiety and depression scores of the participants (*N* = 369)

Sl. no.	Knowledge	*N* (%)	DASS stress		DASS anxiety		DASS depression	
			Mean ± SD	*p* value	Mean ± SD	*p* value	Mean ± SD	*p* value
1.	Knowledge of agents that can effectively kill the virus causing COVID							
	56° for 30 min, diethyl ether, 75% ethanol, peracetic acid and other lipid solvents	146 (39.6)	11.97 ± 10.30	0.88	11.05 ± 9.60		9.73 ± 10.90	0.01*
	Don’t know	224 (60.4)	12.27 ± 10.30		10.60 ± 9.60		12.73 ± 10.90	
*2.	Knowledge of incubation period of COVID-19							
	Usually 3–7 days, with a maximum of 14 days	335 (90.8)	12.03 ± 10.29	0.47	10.72 ± 9.60	0.71	11.61 ± 10.90	0.73
	Don’t know	34 (9.2)	13.35 ± 10.40		11.35 ± 9.67		10.94 ± 11.03	
3.	Knowledge of main mode of transmission of COVID-19							
	Through respiratory droplets such as coughing and sneezing	347 (94.4)	12.03 ± 10.29	0.36	10.44 ± 9.60	0.007*	11.32 ± 10.90	0.12
	Don’t know	22 (5.6)	14.09 ± 10.49		16.09 ± 9.75		15.00 ± 11.11	
4.	Knowledge of other mode of transmission of COVID-19							
	Through contact with contaminated surface and subsequently touching face	345 (93.5)	11.75 ± 10.30	0.004*	10.38 ± 9.60	0.002*	11.60 ± 10.90	0.97
	Don’t know	24 (6.5)	18.00 ± 10.49		16.50 ± 9.60		10.67 ± 11.03	
5.	Knowledge regarding preventive measures for COVID-19?							
	By use of face mask, social distancing, and hand washing	349 (94.6)	12.09 ± 10.30	0.64	10.64 ± 9.60	0.24	11.72 ± 10.90	0.19
	Don’t know	20 (5.4)	13.20 ± 10.37		13.20 ± 9.67		8.50 ± 10.98	
6.	Knowledge of early general symptoms of COVID-19							
	Fever, fatigue, dry cough, and gradually breathlessness	238 (91.6)	11.58 ± 10.30	0.71	10.19 ± 9.60	0.13	11.40 ± 10.90	0.31
	Don’t know	31 (8.4**)**	12.30 ± 10.38		12.90 ± 9.67		8.52 ± 10.97	
7.	Knowledge of symptoms in severe cases of COVID-19							
	Severe breathlessness with low blood oxygen	317 (85.9)	11.75 ± 10.30	0.06	10.66 ± 9.60	0.54	11.56 ± 10.90	0.78
	Don’t know	52 (14.1)	14.62 ± 10.40		11.54 ± 9.67		11.42 ± 10.90	
8.	Knowledge that COVID-19 can also present with gastrointestinal symptoms							
	Yes, such as diarrhea, vomiting and abdominal pain	274 (74.3)	13.05 ± 10.31	0.005*	11.51 ± 9.61	0.01*	11.64 ± 10.90	0.77
	Don’t know	95 (25.7)	9.56 ± 10.90		8.65 ± 9.66		11.26 ± 10.98	
9.	Knowledge of effect of psychological balance on body’s immunity							
	Yes, improves body’s immunity	287 (77.8)	12.38 ± 10.31	0.43	11.18 ± 9.61	0.13	11.38 ± 10.90	0.59
	Don’t know	82 (22.2)	11.36 ± 10.31		9.37 ± 9.60		12.12 ± 10.90	

* means statistically significant association between the concerned variables

## Discussion

Previous studies in Saudi Arabia have shown a high prevalence of anxiety and depression in medical students [23], and college students [24] irrespective of any emergency or epidemic. Studies worldwide have shown an increased psychological impact on college and university students during the “COVID-19” pandemic [14–17]. Overall, we found higher stress, anxiety, and depressive symptoms in the study participants when compared to previous studies in the region of Saudi Arabia [25, 26]. Contrary to this, few studies found higher level of stress than our study participants [27, 28]. The higher prevalence in our sample could be because of the fact that our sample consisted of purely university students and female participants which is in line with previous studies reporting a higher prevalence of anxiety and depression in female gender and university students [8–10]. Moreover, we found significant number of subjects with positive past and, family history of psychiatric illness respectively, which could be another reason for higher prevalence as compared to previous studies. Nonetheless, factors like coping styles, social support [29], personality [30] and religiosity [31] could be among other factors affecting the psychological heath of students. Interestingly, depression, anxiety, and stress have not only been highly prevalent during the “COVID-19” pandemic, but also after the movement lockdown was lifted [32]. Our study indicates that university students are having a considerable amount of mental health burden. However, these findings may be confounded by the time and type of examination in different years, different studying materials and different studying years. The ambiguity about the future and detrimental effect on academic progression could be the cause for mental health issues among students.

Participants getting infected with SARS-CoV-2 irrespective of admission showed significantly high levels of depression and anxiety while those admitted to hospital and ICU reported significantly high levels of anxiety and stress. This finding is similar to studies reporting a high level of anxiety, depression and, PTSD in patients diagnosed with “COVID-19” [33]. Nonetheless, coronavirus infections are also known as neurotropic viruses due to their affinity to the nervous system [34]. SARS-CoV-2 neuro-invasion was demonstrated in human cell cultures and post-mortem studies [35]. Thus, the neuroinflammation caused by SARS-CoV-2 may cause neuropsychiatric illness [36].

It was found that most of the participants had good knowledge (90–95%) about “COVID-19” for most of the knowledge variables when compared to a Chinese study (50–60%) [37]. The knowledge regarding the ability of solvents like diethyl ether, 75% ethanol, peracetic acid, other lipid solvents and, 56 °C heat effectively killing the virus causing “COVID-19” was found in 39.6%, which is comparable to the finding of a Chinese study reporting 30% participants knowing the same factor [37]. Our study found that 77.8% of the participants knew that psychological balance improves immunity, whereas a similar study from China revealed only 37% of participants knew about this fact [37]. Our findings suggest that knowledge regarding the incubation period, preventive measures, early signs of “COVID-19” and, the effect of psychological balance on immunity was not s depression, anxiety and, stress symptoms. Similarly, previous studies have suggested that overall knowledge about “COVID 19” had a protective psychological effect [38–40]. A previous study reported a higher level of anxiety and depression in individuals having good knowledge about handwashing as a preventive measure for “COVID-19” [32], whereas our study reported high levels of anxiety and stress in individuals knowing about surface contamination and respiratory droplet as a mode of spread. Research and clinical observations suggested that during pandemics increased anxiety and stress in association with fear of coming in contact with infected objects or surfaces [40]. However, descriptive findings of this study indicate that university students possessed an adequate level of knowledge related to “COVID-19” at the time of the survey administration. Additionally, our sample included students from medical and paramedical courses hence, more likely to be attuned to correct information related to “COVID-19”.

Adequate pandemic-related knowledge, teaching psychological strategies (e.g., training of resilience factors and coping strategies) and social support help reduce negative psychological affect [41–43]. Besides, biofeedback, mindfulness techniques (e.g., meditation) [42] and cognitive behavioral therapy [44] are useful measures to combat psychological affect of “COVID-19”. Online psychological counselling services by mental health professionals in medical institutions, universities, and academic societies and online psychological self-help intervention systems, including online cognitive behavioral therapy for depression, anxiety, and insomnia have shown to be effective in combating the mental health issues [45].

### Limitations

Our study had few limitations worth mentioning. Since the study design is cross-sectional, the causal association cannot be established. Thus, findings need to be supported by well-designed longitudinal studies. Further studies about the impact of COVID-19 with follow-up these students may help shed some light on the psychological effect among students. Since the data collection was online, the reliability and accuracy of information provided may be limited. Furthermore, the recruitment of subjects via social media could also induce a selection bias of those students that experience more than average mental health issues. There is a possibility of self-rating bias and issues of subjectivity and reliability since the measure used to assess psychological impact was a self-rating scale. The higher scores were not further evaluated by a psychiatrist to confirm the presence of a psychiatric disorder. Moreover, the questionnaire and scale were not translated in Arabic.

## Conclusion

Our study findings reflect the increased mental health burden among the university students due to the “COVID-19” pandemic. The Government should consider incorporating mental health and psychological intervention within the “COVID-19” outbreak prevention and mitigation program for vulnerable groups such as health care professionals and students. To alleviate the mental health burden of the “COVID-19” pandemic, mental health interventions and professionally trained counsellors shall be made available within the campus. Future research should be aimed at targeting multiple universities for longer periods to identify the full spectrum and course of student mental disorders. Longitudinal studies in the future may help answer whether direct or indirect exposure to the “COVID-19” virus added to mental health disorders. Continuation of face-to-face academic activities and social interaction may be of paramount importance to protect the psychological wellbeing of university students.

## Data Availability

Not applicable.

## Abbreviations

COVID-19: Coronavirus disease 2019
WHO: World Health Organization
PTSD: Post-traumatic stress-disorder
DASS-21: Depression Anxiety Stress Scale-21

## References

[1] Saudi Ministry of Health; 2020. Available from: https://covid19.moh.gov.sa/. Accessed 1 Dec 2020 [Ref list].

[2] Wang C, Pan R, Wan X, Tan Y, Xu L, Ho CS, Ho RC (2020). Immediate psychological responses and associated factors during the initial stage of the 2019 coronavirus disease (COVID-19) epidemic among the general population in China. Int J Environ Res Public Health.

[3] Li J, Yang Z, Qiu H (2020). Anxiety and depression among general population in China at the peak of the COVID-19 epidemic. World Psychiatry.

[4] Lei L, Huang X, Zhang S, Yang J, Yang L, Xu M (2020). Comparison of prevalence and associated factors of anxiety and depression among people affected by versus people unaffected by quarantine during the COVID-19 epidemic in Southwestern China. Med Sci Monit.

[5] Qiu J, Shen B, Zhao M, Wang Z, Xie B, Xu Y (2020). A nationwide survey of psychological distress among Chinese people in the COVID-19 epidemic: implications and policy recommendations. Gen Psychiatry.

[6] González-Sanguino C, Ausín B, Castellanos MA (2020). Mental health consequences during the initial stage of the 2020 coronavirus pandemic (COVID-19) in Spain. Brain Behav Immun.

[7] Osman DM, Khalaf FR, Ahmed GK (2022). Worry from contracting COVID-19 infection and its stigma among Egyptian health care providers. J Egypt Public Health Assoc.

[8] Al-Hanawi MK, Mwale ML, Alshareef N, Qattan AMN, Angawi K, Almubark R (2020). Psychological distress amongst health workers and the general public during the COVID-19 pandemic in Saudi Arabia. Risk Manag Healthc Policy.

[9] Alyami HS, Naser AY, Dahmash EZ, Alyami MH, Alyami MS. Depression and anxiety during the COVID-19 pandemic in Saudi Arabia: A cross-sectional study. Int J Clin Pract. 2021;75(7):e14244. 10.1111/ijcp.14244. 10.1111/ijcp.14244 PMC8249996 33876864

[10] Alkhamees AA, Alrashed SA, Alzunaydi AA, Almohimeed AS, Aljohani MS (2020). The psychological impact of COVID-19 pandemic on the general population of Saudi Arabia. Compr Psychiatry.

[11] Zimmermann M, Bledsoe C, Papa A (2021). Initial impact of the COVID-19 pandemic on college student mental health: a longitudinal examination of risk and protective factors. Psychiatry Res.

[12] Rose S (2020). Medical student education in the time of COVID-19. JAMA.

[13] Heinen I, Bullinger M, Kocalevent RD (2017). Perceived stress in first year medical students—associations with personal resources and emotional distress. BMC Med Educ.

[14] Cao W, Fang Z, Hou G, Han M, Xu X, Dong J, Zheng J (2020). The psychological impact of the COVID-19 epidemic on college students in China. Psychiatry Res.

[15] Wang ZH, Yang HL, Yang YQ, Liu D, Li ZH, Zhang XR (2020). Prevalence of anxiety and depression symptom, and the demand for psychological knowledge and interventions in college students during COVID-19 epidemic: a large cross-sectional study. J Affect Disord.

[16] Tanga W, Hu T, Hu B, Jin C, Wang G, Xie C (2020). Prevalence and correlates of PTSD and depressive symptoms one month after the outbreak of the COVID-19 epidemic in a sample of home quarantined Chinese university students. J Affect Disord.

[17] Abdulghani HM, Sattar K, Ahmed T, Akram A (2020). Association of COVID-19 pandemic with undergraduate medical students’ perceived stress and coping. Psychol Res Behav Manag.

[18] Voltmer E, Kötter T, Spahn C (2012). Perceived medical school stress and the development of behavior and experience patterns in German medical students. Med Teach.

[19] Husky MM, Kovess-Masfety V, Swendsen JD (2020). Stress and anxiety among university students in France during Covid-19 mandatory confinement. Compr Psychiatry.

[20] Galic M, Mustapic L, Šimunic A, Sic L, Cipolletta S (2020). COVID-19 related knowledge and mental health: case of Croatia. Front Psychol.

[21] Lovibond SH, Lovibond PF (1995). Manual for the Depression Anxiety Stress Scales.

[22] Antony MM, Cox BJ, Enns MW, Bieling PJ, Swinson RP (1998). Psychometric properties of the 42-item and 21-item versions of the depression anxiety stress scales in clinical groups and a community sample. Psychol Assess.

[23] Inam B (2007). Anxiety and depression among students of a medical college in Saudi Arabia. Int J Health Sci.

[24] Amr M, Amin TT, Saddichha S, Al Malki S, Al Samail M, Al Qahtani N (2013). Depression and anxiety among Saudi University students: prevalence and correlates. Arab J Psychiatry.

[25] Khoshaim HB, Al-Sukayt A, Chinna K, Nurunnabi M, Sundarasen S, Kamaludin K, Baloch GM, Hossain SFA (2020). Anxiety level of university students during COVID-19 in Saudi Arabia. Front Psychiatry.

[26] Al-Hazmi AM, Sheerah HA, Arafa A (2021). Perspectives on telemedicine during the era of COVID-19; What can Saudi Arabia do?. Int J Environ Res Public Health.

[27] AlAteeq DA, Aljhani S, AlEEsa D (2020). Perceived stress among students in virtual classrooms during COVID-19 outbreak in KSA. J Taibah Univ Med Sci.

[28] Badr AF, Binmahfouz LS (2020). Impact of COVID-19 pandemic on mental health among pharmacy students at King Abdulaziz University, Jeddah, Saudi Arabia. Mediterr J Soc Sci.

[29] Akbar Z, Aisyawati MS (2021). Coping strategy, social support, and psychological distress among university students in Jakarta, Indonesia during the COVID-19 pandemic. Front Psychol.

[30] Liua S, Lithopoulosa A, Zhanga CQ, Garcia-Barrerad MA, Rhodesa RE (2021). Personality and perceived stress during COVID-19 pandemic: testing the mediating role of perceived threat and efficacy. Pers Individ Differ.

[31] Lucchetti G, Garcia Góes L, Amaral SG, Ganadjian GT, Andrade I, Almeida PO, Mendes do Carmo V, Gonzalez Manso ME (2020). Spirituality, religiosity and the mental health consequences of social isolation during Covid-19 pandemic. Int J Soc Psychiatry.

[32] Woon LS-C, Leong Abdullah MFI, Sidi H, Mansor NS, Nik Jaafar NR (2021). Depression, anxiety, and the COVID-19 pandemic: severity of symptoms and associated factors among university students after the end of the movement lockdown. PLoS ONE.

[33] Guo Q, Zheng Y, Shi J, Wang J, Li G, Li C (2020). Immediate psychological distress in quarantined patients with COVID-19 and its association with peripheral inflammation: a mixed-method study. Brain Behav Immun.

[34] Reiss CS (2008). Neurotropic viral infections.

[35] Song E, Zhang C, Israelow B, Lu-Culligan A, Sprado A, Skriabine S (2021). Neuroinvasion of SARS-CoV-2 in human and mouse brain. J Exp Med.

[36] Vuren EJV, Steyn SF, Brink CB, Moller M, Viljoen FP, Harvey BH (2021). The neuropsychiatric manifestations of COVID-19: interactions with psychiatric illness and pharmacological treatment. Biomed Pharm.

[37] Liang L, Ren H, Cao R, Hu Y, Qin Z, Li C, Mei S (2020). The effect of COVID-19 on youth mental health. Psychiatr Q.

[38] Tee ML, Tee CA, Anlacan JP, Aligam KJG, Reyes PWC, Kuruchittham V, Ho RC (2020). Psychological impact of COVID-19 pandemic in the Philippines. J Affect Disord.

[39] Kecojevic IA, Basch CH, Sullivan M, Davi NK (2020). The impact of the COVID-19 epidemic on mental health of undergraduate students in New Jersey, cross-sectional study. PLoS ONE.

[40] Taylor S (2019). The psychology of pandemics: preparing for the next global outbreak of infectious disease.

[41] Kunzler AM, Stoffers-Winterling J, Stoll M, Mancini AL, Lehmann S, Blessin M (2021). Mental health and psychosocial support strategies in highly contagious emerging disease outbreaks of substantial public concern: a systematic scoping review. PLoS ONE.

[42] Hatta S (2020). The psychological sequelae during mental health and Covid-19 pandemic: learning from the past for today’s coping styles. Med Health.

[43] Leppin AL, Bora PR, Tilburt JC, Gionfriddo MR, Zeballos-Palacios C, Dulohery MM (2014). The efficacy of resiliency training programs: a systematic review and meta-analysis of randomized trials. PLoS ONE.

[44] Cole CL, Waterman S, Hunter ECM, Bell V, Greenberg N, Rubin GJ (2020). Effectiveness of small group cognitive behavioural therapy for anxiety and depression in Ebola Treatment Centre staff in Sierra Leone. Int Rev Psychiatry.

[45] Liu S, Yang L, Zhang C, Xiang Y, Liu Z, Hu S (2020). Online mental health services in China during the COVID-19 outbreak. Lancet Psychiatry.

